# How to alter path dependency and promote the use of EPC model in public projects of China?

**DOI:** 10.1371/journal.pone.0266957

**Published:** 2022-04-19

**Authors:** Shaowen Wang, Xiaojun Liu, Na Liu

**Affiliations:** School of Management, Xi’an University of Architecture and Technology, Xi’an, Shaanxi, China; Shenzhen University, CHINA

## Abstract

The key to promoting the EPC (Engineering, Procurement, Construction) model in China’s public construction projects is to alter the path dependence of a project owner’s choice of project delivery model (PDM). This study uses evolutionary game theory to discuss the mechanism of government incentives as an external motivation to alter path dependence in the PDM. In addition, a cellular automata simulation to examine the influence of various government incentives on the project owner’s choice. The results show that the combination of subsidies and penalties can produce the best incentive. Subsidies are more effective at promoting PDM institutional change, whereas penalties are more effective at preventing PDM institutional change from anti-recession effects. Based on our results, we propose that the Chinese government should take active subsidy measures at the initial stage of EPC promotion, and adopt a dynamic incentive strategy of continuously reducing subsidies and increasing penalties according to the improvement of the development degree of EPC model.

## Introduction

The EPC model has been used in public construction projects invested by the government in more and more countries, and has achieved good performance [1]. In the past two decades, the use of EPC model instead of DBB model which in the order of "design, bid, then build" in public projects has become the main practice in developed countries such as the United Kingdom, the United States and the European Union [2–5]. At present, the Chinese government is actively promoting the project general contracting mode, and takes it as one of the key tasks of China’s construction industry reform. Especially in 2016, the “Opinions of the Ministry of Housing and Urban-Rural Development on Further Promoting the General Contracting Development of Projects” stated that it is necessary to deepen the reform of the organization and implementation of construction projects as well as to promote the EPC model. Construction units should give priority to adopting the EPC model, and government investment projects and assembly buildings should actively adopt the EPC model. However, data from China public service platform for tendering and bidding show that the number of EPC projects in China is increasing year by year (286, 1290, 4825, 8098, 10545, 12109 respectively from 2016 to 2021), but the growth rate is very slow (from 2016 to 2021, the proportion of EPC projects in the total number of projects is 0.374%, 0.533%, 1.203%, 1.726%, 2.448%, 2.338%). Therefore, how to effectively promote the development of EPC model in public projects has become a challenge facing the Chinese government and the construction industry.

Previous research has shown that it is difficult to promote the EPC model in public projects [6]. Kent and Becerik-Gerber [7] pointed out that the reason for the slow development of the EPC model was that the construction industry accustomed to traditional leadership methods, responsibilities and opportunities. Bygballe and Sward [8] believed that the existing system logic and industry conservatism restrict the evolution of the EPC model. Azhar [9] showed that legal, organizational, and technical issues were the constraints to the use of alternative PDM in public projects. Asmar [10] showed that an important challenge in the transition from DBB model to EPC model was the pursuit of the lowest cost to the best value. Wang and Liu [11] pointed out that public construction projects have great influence and are usually subject to strict supervision and laws and regulations. These studies seem to show that path dependence in institutional change is the main reason restricting the use of the EPC Model in public projects. However, there is no research on how to break the path dependence of PDM from the perspective of institutional change. Therefore, this study takes the promotion of the EPC model in public project in China as an example, and aims to explore how to alter the path dependence in the PDM selection of public project owners.

### Main theories

#### Path dependence theory

Institutional change is an increasingly popular perspective for analyzing long-term historical developments in firms and industries or referring to other units of analysis [12]. It aids our understanding of how historical choices may have a constraining (or enabling) effect on future institutional changes [13].

The theory of institutional change argues that purposeful institutional change does not occur in a vacuum but is strongly guided by the legacies of the past and the existing institutional arrangements that affect current decisions [13]. If the initial system is good, then along the existing path, changes to the system may enter the track of a positive circle and be rapidly optimized [14]. This is called path dependence I. Otherwise, it may follow the original less optimal path and become stuck in a negative cycle of inefficient institutions, called path dependence II. Obviously, the promotion of the EPC model facing the existence of path dependence II. Path dependence theory holds that once the path dependence of the institutional change is formed, it is difficult to be broken from the inside, and the institutional change cannot be effectively realized, even if the institutional theory after a reform is superior and more efficient [15].

Using path dependence theory can better explain and understand the dilemma faced by the promotion of EPC model in public projects. With the complex changes in the scale of construction projects and the increasing social division of labor, the DBB model was initially adopted by the government and enterprises [16]. To better implement this model requires significant costs, such as the establishment of government management departments, the promulgation of laws and regulations, and the training of construction workers, which promote the self-reinforcement of the DBB model and constitute the initial cost of the engineering transaction model change. The DBB model has gradually become the mainstream PDM and has been in use ever since [17]. Various departments and practitioners in the construction industry are very familiar with the entire process of project construction from project establishment, application for construction, project bidding, construction management to project acceptance, and the total cost of using the DBB model has gradually decreased [18]. Cost reductions will make more enterprises willing to achieve their own purpose [19]. This is a form of self-reinforcing, or positive feedback. The path dependence of the DBB model is thus generated.

Because of the inertia of the DBB model, it would be very difficult to form a new PDM learning development path. Relevant entities and institutional constraints, given the economic rationality assumption, will consolidate their most favorable institutions, and interest organizations or interest groups will form within the entire engineering construction system. The coordinated effect of self-reinforcement provides standardized behavioral guidelines and specifications for all stakeholders in the construction project [20]. All stakeholders will profit by complying with the rules, thereby increasing the recognition and generating universal and strong adaptability [21]. This lays a solid foundation for the persistence of the DBB model and is the root of the current EPC model’s poor operability and its difficulty in implementation. At the same time, the EPC model has not yet formed a mature and stable system specification in terms of law formulation, contract management, and risk response, which increases the uncertainty of engineering construction and further strengthens the application of the DBB model in reverse, forming a “lock-in” path dependence.

### Incentive mechanism

Economists regard such path dependence as market failure and believe that the failure of the “invisible hands” should be corrected by “visible hands”. Many studies have shown that incentive mechanisms are effective for the government to adjust market failures; government subsidies and tax policies are widely used as incentives for new technologies, systems, and behaviors with externalities. Zhou et al. [22] studied the impact of price subsidies and tax incentives on the development of the electric vehicle industry through the game of enterprises and consumers and considered that dynamic incentives and tax incentives are more effective. They also analyzed the role of fiscal policy in third-party environmental pollution treatment through a three-party evolutionary game model of local government, polluting enterprises, and third-party enterprises. Wang and Shi [23] constructed an evolutionary game model for industrial pollution between local governments and enterprises to study the evolutionary stable strategy under incentive penalties. Sun et al. [24] discussed the evolutionary stability strategies of suppliers and manufacturers given government subsidies. Wang et al. [25] showed the introduction of government subsidies as a first incentive mechanism to be helpful in promoting the process of logistics market greening. Liu et al. [26] showed that government subsidies have a positive promotional effect on research and development.

Therefore, external motivation has become an important factor in achieving institutional change. Government incentives are an important policy tool, including subsidy measures and punitive measures, which can provide external power for the institutional change of PDM [27]. In the construction industry, the incentive mechanism has also proved to be an effective measure in the promotion of BIM technology [28], green building [26,29] and construction industrialization [30].

In summary, the existence of path dependence in PDM selection is the main reason that restricts the use of EPC mode in public projects in theory, and incentive measures can be used as an external motivation to alter this path dependence which is also theoretically feasible. Therefore, this study hypothesizes that government incentives can effectively alter the path dependence in PDM selection, thereby promoting the use of the EPC model. The remainder of the paper is organized as follows. In Section 3, we build an evolutionary game model of the project owner s choice of PDM under government incentives that have been used to reveal the mechanism of the project owner’s choice of PDM, and use cellular automata to simulate the impact of different government incentives on the choice of project owners. Section 4 analyzes the equilibrium point of the evolutionary game model and the mechanism of government incentives on the choice of project owners. Section 5 discusses the stability and anti-recession standards of PDM evolution, points out the dynamic adjustment of incentives, and summarizes the contributions of this paper. Section 6 presents conclusions and recommendations.

## Methods

### Evolutionary game theory

Aoki [31] believes that evolutionary game theory is more suitable for analyzing institutional evolution, which comes in the form of conventions and customs. Greif [32] combined game theory with path dependence analysis to open new perspectives for the study of institutional change and selection. The theory of evolution borrowed and extended from biology provides a useful framework for the analysis of institutional change; its key processes are mutation, selection, and regeneration. Therefore, the evolutionary game is a suitable analysis method.

Evolutionary game theory uses groups of individuals with limited rationality as the research object [33]. It believes that individual decisions are achieved through dynamic processes such as imitation, learning, and mutation among individuals. Limited rationality, learning mechanisms, and decision-making processes better comport with reality [27]. Evolutionary game theory combines game theory with dynamic evolution analysis. It contends that when the economy is in a stable equilibrium state, economic subjects with limited rationality will continuously imitate and learn favorable strategies, and eventually form stable strategies, that is, an evolutionary stability strategy (ESS) [34]. It can provide a good dynamic explanation for the institutional changes of the EPC model [22]. In addition, evolutionary games still set up a payoff matrix objectively and can be analyzed via expected utility theory [35].

In China, DBB mode is the most widely existing PDM, and EPC mode is the PDM encouraged by the government. This study hypothesizes that a small group using the EPC model appears in the large group using the DBB model and forms a mixed group. When the benefits of the small group of the EPC model in the mixed group become greater than the benefits of the DBB model in the original group, the promotion of the EPC model will alter the path dependence and become widely adopted.

Before constructing the game model, we must provide the following assumptions to ensure the objectivity and scientific nature of the evolutionary game model [27,35].

1. The government is an important maker, guide, and propagandist of the EPC model promotion policy. It seeks to maximize the overall social benefits by rewarding project owners who choose the EPC model and punishing project owners who choose the DBB model.
2. Both project owner A and owner B are rational market entities, maximizing their own interests.
3. There are DBB and EPC mode strategies. The probability of project owner A choosing EPC mode is p, and the probability of choosing DBB mode is 1-p. The probability of project owner B choosing EPC mode is q, and the probability of DBB mode is 1-q.
4. When project owner A and owner B simultaneously choose the DBB mode, the total revenue is F, and then the total revenue obtained by project owner A and owner B choosing the EPC mode simultaneously is F+αF−βF. αF is the total revenue increased to the project owner by choosing the EPC model (obtained by saved engineering management costs, reduced construction period costs, and improved overall project performance); βF is the total cost increased to the project owner by choosing EPC modes (obtained by wasted learning costs of the DBB model, increased learning costs and adaptation costs of implementing the EPC model); (0<*α*<*β*)
5. When project owner A chooses the DBB model and project owner B chooses the EPC model, the possible penalty for project owner A is γF, the possible subsidy for project owner B is δF; (0≤*γ*,*δ*).

Therefore, the Nash equilibrium of this game depends on the relative sizes of *α*, *β*, *γ*, *δ*. Based on the above assumptions, we construct the project owner A-owner B payoff matrix as shown in Table 1 [22].

**Table 1:** Payoff matrix of project Owner A-Owner B.

Owner A	Owner B	
	EPC (q)	DBB(1−q)
EPC(p)	F+αF−βF,F+αF−βF	F+αF−βF+δF,F−γF
DBB(1−p)	F−γF,F+αF−βF+δF	*F*,*F*

### Cellular automata (CA) simulation

The cellular automaton (CA) has simplified the construction of some models. Because it can grasp the essential characteristics of system evolution, which can reflect complex evolutionary behavior, it is an important research tool for simulating complexity or complex systems. The method has received widespread attention in the natural and social sciences. Cellular automata simulation differs, however, from traditional simulation methods. Its basic principle is to use a parallel evolution of many cells under simple rules to simulate complex phenomena. It is a good behavioral simulation tool commonly used in human cooperation relationship changes, social dynamics and changes in human concepts [36]. In our study employing CA to simulate project owner behavior can help design incentive mechanisms.

**Fig 1:**
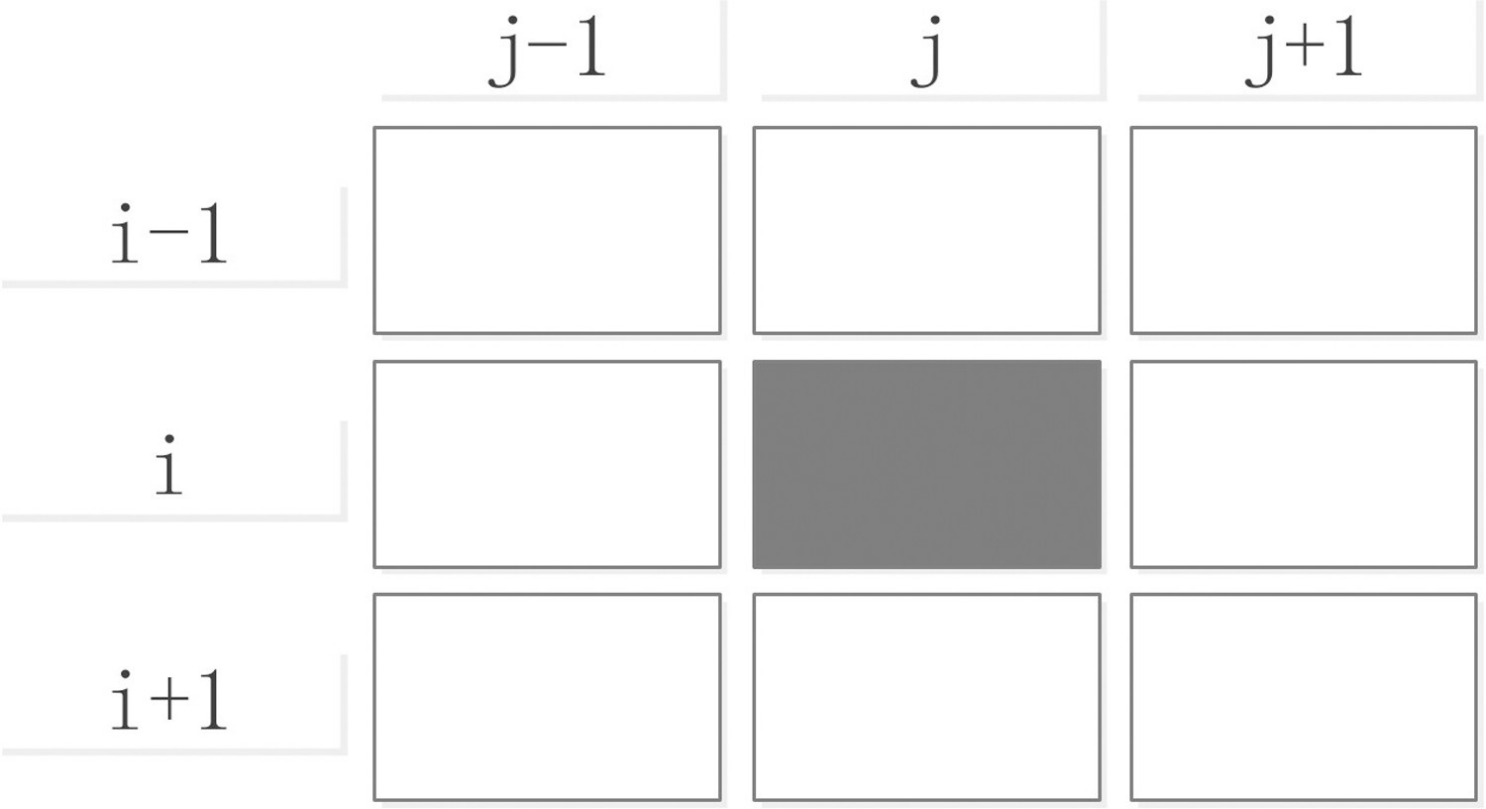
Moore-type neighbor cell.

CA is composed of five elements: cell, cell space, neighbor, cell state, and state evolution rules [37]. The project owner behavior model is established as follows:

1. Cells. Represented by a cell in a square checkerboard grid of 30×30; that is, a cell represents a project owner [38].
2. Cell space. Cell space represents the set of project owners and is represented by a set of 30×30 square checkerboard grids.
3. Neighbor. The neighbor cell is a set of cells that will affect the central cell at the next moment. This study chooses a Moore-type neighbor cell, that is, eight adjacent cells around the cell are its neighbor cells, as shown by the gray in Fig 1. The cell is the Moore-type neighbor of the central black cell.

4) Cell state. The state of the cell is the value of a certain aspect of the cell under investigation. In this study, there are three states of the automaton cell individual at each moment, that is *N* = {−1,0,1}.
  $N_{ij}^{t}=1$ indicates that the project owner chooses the EPC model.
  $N_{ij}^{t}=0$ indicates that the project owner without an explicit attitude chooses the EPC model or DBB model without any tendency.
  $N_{ij}^{t}=-1$ indicates that the project owner chooses the DBB model.
5) State evolution rules. The state evolution rule is a dynamic function that determines the state of the cell at the next moment according to the current state and the state of its neighbors. According to the principle of cellular automata, the state of the cellular cell at the next moment is affected by two factors: the behavior of the neighbor’s project owner, and the herd mentality. Let the corresponding probability of herd mentality be *ω*. When it is not affected by external factors, the current cell will use this probability to decide to change to the behavior of neighbor cells.

## Results

### Evolutionary equilibrium analysis

According to the basic assumptions, the specific game model is constructed as follows:

1) The overall expectations for strategic of project owner A:

$$U_{A(EPC)}=q(F+\alpha F-\beta F)+(1-q)(F+\alpha F-\beta F+\delta F)$$ (1)

$$U_{A(DBB)}=q(F-\gamma F)+(1-q)F$$ (2)

$$\bar{U_{A}}=pU_{A(EPC)}+(1-p)U_{A(DBB)}$$ (3)

Therefore, the replicator’s dynamic equation about project owner A’s game behavior of choosing the EPC model is:

$$F(p)=\frac{d_{p}}{d_{t}}=p(U_{A(EPC)}-\bar{U_{A}})=p(1-p)[(\alpha -\beta +\delta )-(\delta -\gamma )q]F$$ (4)

According to the replicator dynamic equation, when we let F(p) = 0, it is not difficult to find three possible steady-state points:

$$p_{1}^{*}=0,\;p_{2}^{*}=1,\;q^{*}=\frac{\alpha -\beta +\delta }{\delta -\gamma }$$ (5)

The same calculations give the strategic expectations of project owner B:

$$U_{B(EPC)}=p(F+\alpha F-\beta F)+(1-p)(F+\alpha F-\beta F+\delta F)$$ (6)

$$U_{B(DBB)}=p(F-\gamma F)+(1-p)F$$ (7)

$$\bar{U_{B}}=qU_{B(EPC)}+(1-q)U_{B(DBB)}$$ (8)

Therefore, the replicator’s dynamic equation about project owner B’s game behavior of choosing DBB model is:

$$F(q)=\frac{d_{q}}{d_{t}}=q(U_{B(EPC)}-\bar{U_{B}})=q(1-q)[(\alpha -\beta +\delta )-(\delta -\gamma )p]F$$ (9)

Then, the three possible steady-state points for project owner B are:

$$q_{1}^{*}=0,\;q_{2}^{*}=1,\;p^{*}=\frac{\alpha -\beta +\delta }{\delta -\gamma }$$ (10)

The equilibrium points in the evolutionary game between project owner A and owner B are:

$$A\;(1,\;0),\;C\;(0,\;1),\;O\;(0,\;0),\;B\;(1,\;1),\;D[\frac{\alpha -\beta +\delta }{\delta -\gamma },\;\frac{\alpha -\beta +\delta }{\delta -\gamma }]$$ (11)

### Cellular automata simulation results

It can be seen from the foregoing that, because the project owner is rational, the probability of choosing the DBB model must be greater than the probability of without an explicit attitude. In order to explore the impact of different incentive measures on the promotion of the EPC model, the initial state of the government without adopting any incentive measures was first investigated in this study. The initial state was randomly generated with a grid of 30×30 cells. The project owners who chose the EPC model were defined as green; the project owners without an explicit attitude were defined as blue; the project owners who chose the DBB model were defined as red; with k, the number of project owners who chose the EPC model. The simulation evolution rules are as follows:

a. If the neighbor chooses the EPC mode at time t, then at t+1, the probability that the project owner chooses the EPC mode is p, the probability that the project owner chooses the DBB mode is 2(1−*p*)/3, and the probability of no explicit attitude is (1−*p*)/3.
b. If the neighbor’s attitude is not explicit at time t, then at time t+1, the probability that the project owner chooses the EPC mode is (1−*p*)/3, the probability that the project owner chooses the DBB mode is 2(1−*p*)/3, and the probability of no explicit attitude is p.
c. If the neighbor selects the DBB mode at time t, then at t+1, the probability that the project owner chooses the EPC mode is (1−*p*)/3, the probability that the DBB mode is selected is p, and the probability of no explicit attitude is 2(1−*p*)/3.

This study simulates the effect of the change in herd probability on the choice of the project owner. The simulation results show that in the randomly generated model, when the herd probability is *ω* = 0.2, the number of project owners who choose the EPC mode is k = 214. With the increase of *ω*, this value becomes *ω* = 0.4,0.6,0.8, and the number of project owners who choose the EPC mode decreases to k = 181, 116 and 60, respectively. These results show that when the government does not issue any incentive measures, economically rational project owners will follow the herd behavior, and the DBB model will become a wise choice for the project owners, which will eventually lead to PDM’s path dependence entering a “lock-in” state. Therefore, government incentives are necessary to promote the EPC model.

To examine the impact of government incentives on project owner behavior, simulation experiments consider two types of government incentives: subsidies and penalties. The simulation evolution rules of the model are as follows:

d. If the government implements subsidies for the neighbors who choose the EPC mode at time t, then at time t+1, the probability that the project owner chooses the EPC mode is *p*+*I_s_*(1−*p*) and the probability of not having an explicit attitude is 2(1−*I_s_*)(1−*p*)/3, the probability of choosing the DBB mode is (1−*I_s_*)(1−*p*)/3, where *I_s_*≥0, representing the strength of government subsidies.
e. If a neighbor does not have an explicit attitude at time t, the government will not give any subsidy or penalty measures, the probability that the project owner chooses the EPC mode is (1−*p*)/3 at time t+1, the probability of not having an explicit attitude is p, and the probability of choosing the DBB mode is 2(1−*p*)/3.
f. If the government imposes a penalty on neighbors who choose the DBB mode at time t, then at time t+1, the probability of the project owner choosing the EPC mode is (1+*P_s_*)(1−*p*)/3, the probability of not having an explicit attitude is 2(1+*P_s_*)(1−*p*)/3, and the probability of choosing DBB is *p*−P*_s_*(1−*p*), where P*_s_*≥0 represents the strength of the government penalty.

**Fig 2:**
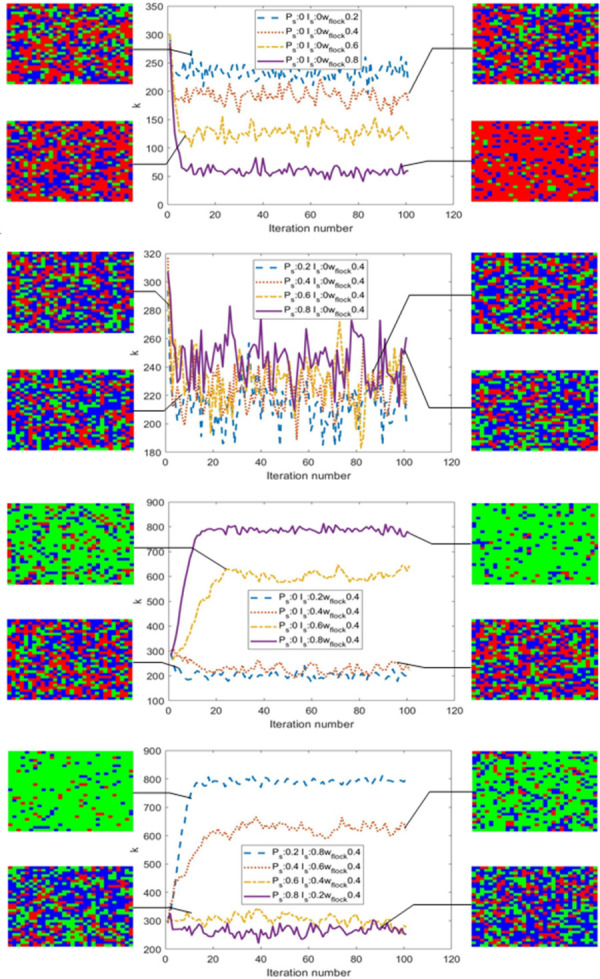
The results of project owner behavior based on cellular automata simulation.

This study discusses the impact of different government incentives on project owners’ behavior, namely: only adopting punitive measures, only adopting subsidy measures, and a combination of subsidy and punitive measures. To avoid the impact of herd behavior on the model and to compare the impact of different government incentives on project owners, the model assumes that the condition *ω* = 0.4 remains unchanged. The simulation results are shown in Table 2, and the results of project owner behavior based on cellular automata simulation are shown in Fig 2.

**Table 2:** Simulation results for different incentives (Data: Author’s).

Case	*ω*	*P_s_*	*I_s_*	k
No incentives	0.2	0	0	214
	0.4	0	0	181
	0.6	0	0	116
	0.8	0	0	60
Only punitive incentives	0.4	0.2	0	201
	0.4	0.4	0	213
	0.4	0.6	0	234
	0.4	0.8	0	261
Only subsidy incentives	0.4	0	0.2	208
	0.4	0	0.4	223
	0.4	0	0.6	643
	0.4	0	0.8	774
Combination of punitive and subsidy incentives	0.4	0.2	0.8	805
	0.4	0.4	0.6	620
	0.4	0.6	0.4	277
	0.4	0.8	0.2	252

The results show that when the government only adopts punitive measures, when the penalty is small (*P_s_* = 0.2), the number of project owners who choose the EPC model (k = 201) is slightly more than that without any incentive measures (k = 181). This indicates that punitive measures are effective, although the effect is small. With each increase in penalty, the number of project owners who choose the EPC model increases, but the effect of the increase is not obvious. As shown in Table 5, we take (*P_s_* = 0.4,0.6,0.8) in order and find that the corresponding number of project owners who choose the EPC mode is k = 213, 234, 261. The results show that the penalty measures can have some incentive effect, but the effect is not significant.

Correspondingly, when the government only implements subsidy measures, and when the subsidy intensity is less (*I_s_* = 0.2), the number of project owners who choose the EPC model is k = 208, which is higher than when there is no incentive (k = 181) or only punitive incentives (k = 201). With an increase in subsidies, the number of project owners choosing the EPC model increases significantly. As shown in Table 5, take (*I_s_* = 0.4,0.6,0.8) in turn, and the corresponding number of project owners who choose the EPC model is k = 223, 643, 774.

The simulation results of the government’s comprehensive incentive measures for subsidies and penalties show that when the penalty is much larger than the subsidy (*P_s_* = 0.8, *I_s_* = 0.2), the number of project owners who choose the EPC model is k = 252. When the penalty is appropriately reduced and the subsidy is increased (*P_s_* = 0.6, *I_s_* = 0.4), the number of project owners who choose the EPC mode increases (k = 277). When the subsidy intensity is slightly greater than the penalty intensity (*P_s_* = 0.4, *I_s_* = 0.6), the number of project owners who choose the EPC model significantly increases (k = 620). When the subsidy is much greater than the penalty (*P_s_* = 0.2, *I_s_* = 0.8), the number of project owners who choose the EPC model is 805. Further analysis found that comprehensively adopting subsidy and penalty incentives can achieve better results than using punitive measures or subsidy measures alone. For example, in the three cases of (*P_s_* = 0.2, *I_s_* = 0.8), (*P_s_* = 0.2) and (*I_s_* = 0.8), the number of project owners who chose the EPC model was 805, 201, and 774, respectively. The results show that the combination of subsidy and penalty measures can achieve the best incentive effect. Among them, the government adopts subsidy incentive measures more effectively than penalty incentive measures.

## Discussion

### Stability and anti-recession standards of PDM evolution

The stability of the equilibrium strategy is derived from the Jacobian matrix [39]. We assume that the Jacobian matrix of the project owner A-owner B game model is J:

$$J=[\begin{array}{cc} (1-2p)[(\beta -\alpha -\gamma )+(\gamma -\delta )q]F & p(1-p)(\gamma -\delta ))F\\ q(1-q)(\gamma -\delta )F & (1-2q)[(\beta -\alpha -\gamma )+(\gamma -\delta )p]F \end{array}]$$ (12)

Then, we obtain:

$$\begin{array}{l} detJ=(1-2p)(1-2q)[(\beta -\alpha -\gamma )+(\gamma -\delta )q][(\beta -\alpha -\gamma )+(\gamma -\delta )p]F^{2}-\\ pq(1-p)(1-q){\left(\gamma -\delta \right)}^{2}F^{2} \end{array}$$ (13)

trJ=(1−2p)[(β−α−γ)+(γ−δ)q]F+(1−2q)[(β−α−γ)+(γ−δ)p]F (14)

Then, the value of the matrix determinant and the trace of the matrix from the five equilibrium points are given in Table 3.

**Table 3:** All parameters.

Game model	Equilibrium Point	detJ / trJ	Expression
Owner A ——Owner B	O (0,0)	detJ	$(\beta -\alpha +\delta )F^{2}$
		trJ	−2(β−α+δ)F
	A (0,1)	detJ	$-(\beta -\alpha -\gamma )(\beta -\alpha -\delta )F^{2}$
		trJ	(*γ*−*δ*)F
	C (1,0)	detJ	$-(\beta -\alpha -\gamma )(\beta -\alpha -\delta )F^{2}$
		trJ	(*γ*−*δ*)F
	B (1,1)	detJ	${\left(\alpha -\beta +\gamma \right)}^{2}F^{2}$
		trJ	−2(α−β+γ)F
	$D(\frac{\alpha -\beta +\delta }{\delta -\gamma },\frac{\alpha -\beta +\delta }{\delta -\gamma })$	detJ	$-\frac{{\left(\alpha -\beta +\delta \right)}^{2}{\left(\beta -\alpha -\gamma \right)}^{2}}{{\left(\delta -\gamma \right)}^{2}}F^{2}$
		trJ	0

According to evolutionary theory, any equilibrium point that satisfies detJ > 0 and trJ < 0 is asymptotically stable, which is deemed an evolutionary stable strategy [11]. If det(J) > 0 and tr(J) > 0, the point is unstable. If det(J) > 0 and tr(J) = 0, the point is defined as a central point. Additionally, if det(J) < 0, the point is defined as a saddle point [40]. As shown in Table 3, the existence of evolutionary stable points (ESS) in the model requires comparing α, β, γ, δ.

As can be seen from the previous section, *α*<*β* and *δ*<*β*−*α*. This is because if *δ*>*β*−*α*, although the project owner will choose the EPC model due to assumed rationality, the government will not be sustainable because of the excessive subsidy. Furthermore, the following three cases are presented in Table 4.

**Table 4:** Stability analysis results.

Game model	Equilibrium Point	DetJ	trJ	Results
Owner A ——Owner B	Case1: *γ*<*δ*<*β*−*α*			
	O (0,0)	+	−	ESS
	A (0,1)	−	−	Saddle point
	B (1,1)	+	+	Unstable point
	C (1,0)	−	−	Saddle point
	$D(\frac{\alpha -\beta +\delta }{\delta -\gamma },\frac{\alpha -\beta +\delta }{\delta -\gamma })$	−	0	Saddle point
	Case2: *δ*<*γ*<*β*−*α*			
	O (0,0)	+	−	ESS
	A (0,1)	−	+	Saddle point
	B (1,1)	+	+	Unstable point
	C (1,0)	−	+	Saddle point
	$D(\frac{\alpha -\beta +\delta }{\delta -\gamma },\frac{\alpha -\beta +\delta }{\delta -\gamma })$	−	0	Saddle point
	Case3: *δ*<*β*−*α*<*γ*			
	O (0,0)	+	−	ESS
	A (0,1)	+	+	Unstable point
	B (1,1)	+	−	ESS
	C (1,0)	+	+	Unstable point
	$D(\frac{\alpha -\beta +\delta }{\delta -\gamma },\frac{\alpha -\beta +\delta }{\delta -\gamma })$	−	0	Saddle point

The above stability analysis shows that in case 1 and case 2, although an evolutionary stability strategy (ESS) has been formed, it is that the project owners A and B do not choose the EPC model, and the institutional change of PDM will not have been realized. It shows that in the case of *γ*<*δ*<*β*−*α* and *δ*<*γ*<*β*−*α*, the project owner’s strategic behavior of choosing the EPC mode is unstable. One possible explanation is that, with fewer penalties and subsidies, the retrogressive effect of institutional changes has occurred. In case3, O (0,0) and B (1,1) are evolutionary stable strategies (ESS); A (0,1) and C (1,0) are unstable points; and $D(\frac{\alpha -\beta +\delta }{\delta -\gamma },\frac{\alpha -\beta +\delta }{\delta -\gamma })$ is a saddle point. Therefore, if the government wants to realize the institutional change of PDM, it is necessary to increase the possibility of B (1,1) as a stable point.

**Fig 3:**
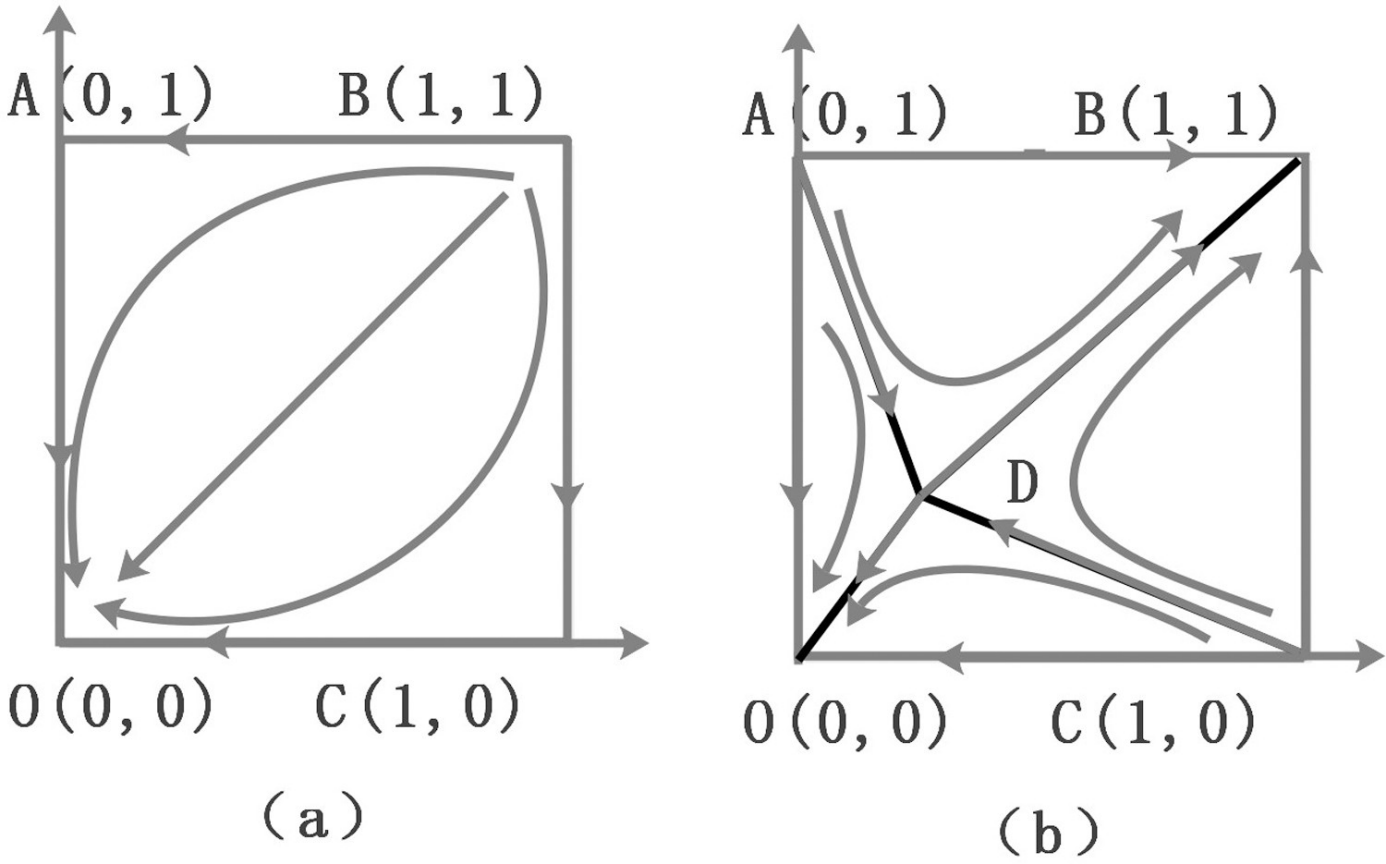
The phase diagram of evolutionary dynamic process.

According to the stability analysis of evolutionary game between project owner A-owner B, the phase diagram of evolutionary dynamic process can be obtained as shown in Fig 3. Case1 and Case2 are shown in (a); and Case 3 is shown in (b).

In Fig 3(b), the three points A, D and C constitute the dividing line of the game converging to different strategies. When the initial state of the behavior game strategy of project owner A-owner B is in the ADCO region, the evolutionary game system will converge to O (0, 0) and the final game result will converge to the stable strategy combination (DBB, DBB). When the initial state of the behavior game strategy of project owner A-owner B is in the ADCB region, the evolutionary game system will converge to B (1, 1) and the final game result will converge to the stable strategy combination (EPC, EPC).

In order to promote the EPC model to the greatest extent and realize the institutional change of PDM, the area of the ADBC needs to be increased, meaning the saddle point D needs to move to the lower left. The probability of convergence to the strategy of (EPC, EPC) increases and the probability of convergence to the strategy of (DBB, DBB) decreases. As is shown in Fig 3, the area of ADCB is: S_ADBC_ = 1-1/2 (p_D_ + q_D_). The four parameters affecting S_ADBC_ are monatomic with S_ADBC_, and their effects on the evolutionary game direction between project owner A-owner B are shown in Table 5.

**Table 5:** The influence of parameter changes on evolutionary game direction.

Parameter change	Saddle point change	Change of phase area and direction of evolution
*β* ↓	p_D_ ↓, q_D_ ↓	S_ADBC_ ↑_,_ (EPC, EPC)
*α* ↑	p_D_ ↓, q_D_ ↓	S_ADBC_ ↑_,_ (EPC, EPC)
*δ* ↑	p_D_↓, q_D_ ↓	S_ADBC_ ↑_,_ (EPC, EPC)
*γ* ↑	p_D_ ↓, q_D_ ↓	S_ADBC_ ↑_,_ (EPC, EPC)

### Dynamic adjustment of incentives

**Fig 4:**
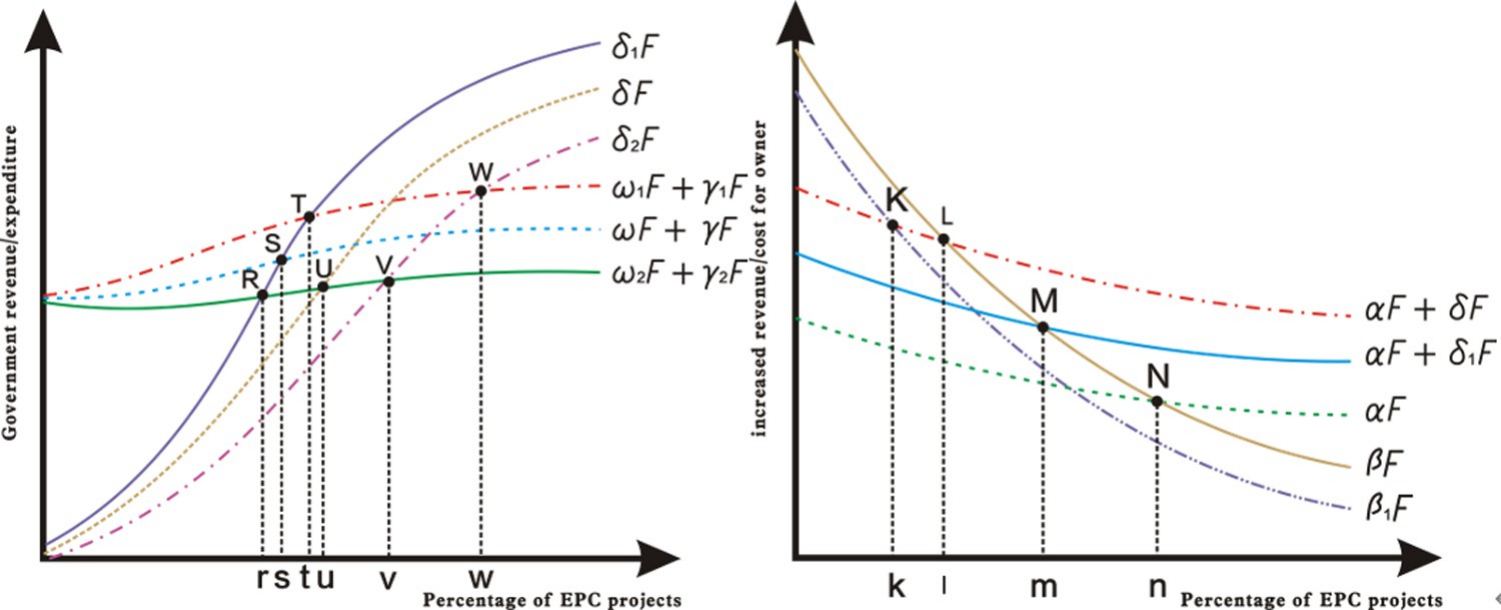
Revenue/Cost analysis for owner or government under different incentive.

According to the theory of diminishing marginal effects, *αF* and *βF* are the reduction function of the number of project owners using the EPC model. As the number of times the project owner uses the EPC model increases, the use of the EPC model increases the total costs (*βF*) and the total benefits (*αF*) to the project owner, which is shown in Fig 4(A). To the left of point N, since the total cost of the EPC model to the project owner is greater than the total revenue, the government needs to give the project owner a subsidy incentive to induce the project owner to choose the EPC model.

From the above analysis, there is an initial government subsidy *δF*, where *δF*<*βF*−*αF*. However, as the number of uses of the EPC mode increases, *δF* = *βF*−*αF* will appear, as shown in point L, which is called the government’s policy adjustment point. At this time, for the project owner, the benefits for choosing the EPC model and the DBB model are the same. If the government does not change the subsidy incentives, the project owners will inevitably continue to choose the EPC model, but the government will damage the overall interests of the society because of the excessive cost of the subsidies. At this time, if the government reduces the subsidy to *δ*_1_, the subsidy can still motivate the project owners. Therefore, the intensity of government subsidies should be dynamically adjusted according to market performance, and it will continue to decrease as the use of the EPC model increases. Therefore, total government expenditure (subsidy) is an increasing function of decreasing slope. The government’s total revenue, including social benefits and penalties, is also an increasing function of EPC use. Since the effect of subsidy measures is better than that of punitive measures, the slope of the total government expenditure curve is greater than the slope of the total return curve, as shown in Fig 4(B). Point S is the point where the government’s revenue and expenditure reach an equilibrium point, also called the government’s policy adjustment point. It is not in the government’s interest to continue to maintain the intensity of subsidies *δ* and penalties *γ*. If the government continues to reduce the subsidy intensity *δ*_2_<*δ*_1_<*δ*, and increase the penalty intensity *γ*_1_>*γ*>*γ*_2_, the government will achieve a break-even point at point V and achieve an EPC project proportion v (v > s). This indicates the direction for the dynamic adjustment of government incentives, that gradually reducing subsidies and increasing penalties can promote the institutional change of PDM to the greatest extent.

## Conclusions

This study takes the promotion of China’s EPC model as an example and uses evolutionary game theory to focus on the path dependence and institutional change of PDM from the perspective of institutional economics. We also respond to DiMaggio’s criticism that scholars are not focusing on the formation of institutions and the mechanisms of institutional change [41]. The results confirms that government incentives can prompt project owners to choose the EPC model, which is an exogenous force that alters the path dependence of PDM. Further research shows that the combined use of subsidies and penalties incentives can achieve better results. Specifically, subsidy measures effectively promote the EPC model, and penalty measures effectively prevent the anti-recession effect of institutional changes. The greater the intensity of subsidies, the easier it is to achieve institutional changes in the PDM, but excessive government subsidies will create a waste of social resources. The greater the penalties, the easier it is for the project owner to stabilize the behavior strategy of choosing the EPC model. Therefore, the government should dynamically adjust the incentive strategy according to market conditions, that is, continuously reduce the subsidy and increase the penalty. The results also provide a plausible explanation for China’s stalled EPC model. That is, if the government does not implement incentive measures, economically rational project owners will act collectively, which will limit the use of EPC models. According to further analysis, when *δ*<*β*−*α*<*γ*, the project owner’s strategy can be stabilized on the EPC model. This provides a standard for anti-recession policies for institutional changes in theory.

According to the research results, we suggest that more attention should be paid to the incentive of subsidy measures in the early stage of promotion of EPC model. With the increase of the proportion of the project owners using the EPC, continuously reducing the subsidy and increasing the penalties can promote the EPC to the maximum extent, and then achieve institutional changes to the PDM. At the same time, rational project owners will make favorable strategic adjustments based on changes in policies and markets. Therefore, we suggest that the government reasonably measure income as well as expenditure and implement precise incentive measures to maximize the effectiveness of incentive policies. In addition, reducing the cost of the EPC model to the project owners and increasing the benefits of the EPC model to the project owners can also increase the possibility of institutional change. This provides guidance for the institutional reform of PDM, that is, in addition to guiding the project owner to choose the EPC model through incentives, it is also important to provide the project owner with an appropriate organization and market environment. For example, the government can improve relevant laws and regulations to reduce the difficulty for construction companies to transform to general contracting business; can improve government supervision functions and create a favorable atmosphere for the promotion and use of the EPC model. Actively cultivating industry talents with the ability to operate in the EPC model, carrying out continuing education, and guiding the direction of talent training in colleges and universities are also possibilities. These measures can greatly reduce the project owner’s learning and adaptation costs using the EPC model. In the future, research should focus on promoting the development of EPC practices.

## Supporting information

S1 File Data. (DOCX)

## References

[1] Choi K, Jung I, Yin Y, Gurganus C, Jeong D. Holistic performance evaluation of highway design-build projects. Journal of management in Engineering. 2020; 36(4): 04020024. doi: 10.1061/(ASCE)ME.1943-5479.0000781

[2] Kebede S D, Tiewei Z. Public work contract laws on project delivery systems and their nexus with project efficiency: evidence from Ethiopia. Heliyon. 2021; 7(3): e06462. doi: 10.1016/j.heliyon.2021.e06462 33763614 PMC7973297

[3] Ahmed S, EI-Sayegh S. Critical review of the evolution of project delivery methods in the coustruction industry. Buildings. 2021; 11(11): 11010011. doi: 10.3390/buildings11010011

[4] Meshref A N, Elkasaby E A, Wageh O. Identifying innovative reliable criteria governing the selection of infrastructures construction project delivery systems. Open Engineering. 2021; 11(1): 269–280. doi: 10.1515/eng-2021-0028

[5] Adamtey S. Cost and time performance analysis of progressive design-build projects. Journal of Engineering, Design and Technology. 2020; 3(19): 686–697. doi: 10.1108/JEDT-05-2020-0164

[6] Demetracopoulou V. O’Brien W J. Khwaja N. Lessons learned from selection of project delivery methods in highway projects: the Texas experience. Journal of Legal Affairs and Dispute Resolution in Engineering and Construction. 2020; 12(1): 04519040. doi: 10.1061/(ASCE)LA.1943-4170.0000340

[7] Kent D C, Becerik-Gerber B. Understanding Construction Industry Experience and Attitudes toward Integrated Project Delivery. Journal of Construction Engineering and Management. 2010; 136(8):815–825. doi: 10.1061/(asce)co.1943-7862.0000188

[8] Bygballe L E, Sward A. Collaborative Project Delivery Models and the Role of Routines in Institutionalizing Partnering. Project Management Journal. 2019; 50(2): 161–176. doi: 10.1177/8756972818820213

[9] Azhar N, Kang Y, Ahmad I U. Factors influencing integrated project delivery in publicly owned construction projects: an information modelling perspective. Procedia Engineering. 2014; 77: 213–221. doi: 10.1016/j.proeng.2014.07.019

[10] Asmar M E, Lotfallah W, Whited G, Hanna A S. Quantitative Methods for Design-Build Team Selection. Journal of Construction Engineering and Management. 2010; 136(8): 904–912. doi: 10.1061/(asce)co.1943-7862.0000194

[11] Wang S, Liu X. Development of EPC model in Chinese public projects: evolutionary game among stakeholders. Journal of Asian Architecture and Building Engineering. Forthcoming 2021. doi: 10.1080/13467581.2021.1971681

[12] Brunninge O, Melander A. The dynamics of path dependence on the individual, organizational and the field levels: MoDo, the Kempe family and the Swedish pulp and paper industry 1873-1990. Management & Organizational History. 2016; 11(2): 189–210. doi: 10.1080/17449359.2016.1150858

[13] Petursson J G, Vedeld P. Rhetoric and reality in protected area governance: institutional change under different conservation discourses in Mount Elgon National Park, Uganda. Ecological Economics. 2017; 131: 166–177. doi: 10.1016/j.ecolecon.2016.08.028

[14] Guo X, Liu X, Chen S, Li L, Fu H. China’s housing provision system: evolution, purchase -rental gap measurement and optimization strategy. Journal of Urban Planning and Development. 2021, 147(4): 04021054. doi: 10.1061/(ASCE)UP.1943-5444.0000766

[15] Heinecke S. The gradual transformation of the Polish public science system. PLOS ONE. 2016; 11(4): e0153260. doi: 10.1371/journal.pone.0153260 27077386 PMC4831804

[16] Minchin R E, Li X, Issa R R, Vargas G G. Comparison of cost and time performance of design-build and design-bid-build delivery systems in Florida. Journal of Construction Engineering and Management. 2013; 139(10): 04013007. doi: 10.1061/(asce)co.1943-7862.0000746

[17] Wang Z, Zhang X. Discussion on EPC project management model. 2013 Fourth International Conference on Intelligent Systems Design and Engineering Applications. 2013; 277–279. doi: 10.1109/isdea.2013.467

[18] Culp G. Alternative project delivery methods for water and wastewater projects: do they save time and money? Leadership and Management in Engineering. 2011; 11 (3):231–240. doi: 10.1061/(asce)lm.1943-5630.0000133

[19] Lee R P, Gloaguen S. Path-dependence, lock-in, and student perceptions of nuclear energy in France: implications from a pilot study. Energy Research & Social Science. 2015; 8: 86–99. doi: 10.1016/j.erss.2015.05.001

[20] Lin Q, Kalantari M, Rajabifard A, Li J. A path dependence perspective on the Chinese cadastral system. Land Use Policy. 2015; 45:8–17. doi: 10.1016/j.landusepol.2015.01.017

[21] Cestino J, Matthews R. A perspective on path dependence processes: the role of knowledge integration in business model persistence dynamics in the provincial press in England. Journal of Media Business Studies. 2016; 13(1): 22–44. doi: 10.1080/16522354.2015.1133785

[22] Zhou X, Zhao R. Cheng L. Min X. Impact of policy incentives on electric vehicles development: a system dynamics-based evolutionary game theoretical analysis. Clean Technologies and Environmental Policy. 2019; 21(5): 1039–1053. doi: 10.1007/s10098-019-01691-3

[23] Wang C, Shi F. An evolutionary game model for industrial pollution management under two punishment mechanisms. International Journal of Environmental Research and Public Health. 2019; 16:2775. doi: 10.3390/ijerph16152775 31382549 PMC6696192

[24] Sun H, Wan Y, Zhang L, Zhou Z. Evolutionary game of the green investment in a two-echelon supply chain under a government subsidy mechanism. Journal of Cleaner Production. 2019; 235:1315–1326. doi: 10.1016/j.jclepro.2019.06.329

[25] Wang Z, Tsai Z, Fu J, Zhao L, Yang L. Internalization of negative external cost of green logistics and incentive mechanism. Advances in Mechanical Engineering. 2017; 9(8):1–12. doi: 10.1177/1687814017715420

[26] Liu M, Liu L, Xu S, Du M, Liu X, Zhang Y. The influences of government subsidies on performance of new energy firms: a firm heterogeneity perspective. Sustainability. 2019; doi: 10.3390/su11174518 .11174518

[27] Zhang S, Wang C, Yu C. The evolutionary game analysis and simulation with system dynamics of manufacturer’s emissions abatement behavior under cap-and-trade regulation. Applied Mathematics and Computation. 2019; 355: 343–355. doi: 10.1016/j.amc.2019.02.080

[28] Yuan H, Yang Y. BIM adoption under government subsidy: technology diffusion perspective. Journal of Construction Engineering and Management. 2020; 146(1): 04019089. doi: 10.1061/(ASCE)CO.1943-7862.0001733

[29] Zou Y, Zhao W, Zhong R. The spatial distribution of green buildings in China: Regional imbalance, economic fundamentals, and policy incentive. Applied Geography. 2017; 88:38–47. doi: 10.1016/j.apgeog.2017.08.022

[30] Yunus R, Suratkon A, Wimala M, Hamid H A, Noor S R M. Motivational factors on adopting modular coordination concept in industrialized building system (IBS). MATEC Web of Conferences. 2016; 47:04107. doi: 10.1051/matecconf/20164704017

[31] Aoki M. Toward a Comparative Institutional Analysis. Cambridge, MA: MIT Press; 2001.

[32] Greif A. Institutions and the Path to the Modern Economy, Lessons from Medieval Trade. Cambridge, U.K. and NY: Cambridge University Press; 2006.

[33] Zhang L, Xue L, Zhou Y. How do low-carbon policies promote green diffusion among alliance-based firms in China? an evolutionary-game model of complex networks. Journal of Cleaner Production. 2018 Nov 03. pii:S0959-6526(18)33428-0. doi: 10.1016/j.jclepro.2018.11.028

[34] Zhang R, Li J. Impact of incentive and selection strength on green technology innovation in Moran process. PLOS ONE. 2020; 6(15): e0235516. doi: 10.1371/journal.pone.0235516 32603355 PMC7326173

[35] Pi Z, Gao X, Chen L, Liu J. The new path to improve construction safety performance in China: an evolutionary game theoretic approach. International Journal of Environmental Research and Public Health. 2019; 16(13): 1–24. doi: 10.3390/ijerph16132443 31324046 PMC6650957

[36] Stamatovic B, Upadhyay R, Vatin N. Cellular Automata in Modeling of Porous Building Materials and Solis Porous Media. Procedia Engineering. 2015; 117: 655–662. doi: 10.1016/j.proeng.2015.08.227

[37] Fan X. Research on government investment behavior in rural infrastructure construction [dissertation]. Harbin: Harbin Institute of Technology; 2009. China.

[38] Cremene M, Dumitresce D, Cremene L. A strategic interaction model of punishment favoring contagion of honest behavior. PLOS ONE. 2014; 9(1): e87471. doi: 10.1371/journal.pone.0087471 24489917 PMC3904992

[39] Zhou C, Xie H, Zhang X. Does fiscal policy promote third-party environmental pollution control in China? an evolutionary game theoretical approach. Sustainability. 2019; 11(16): 1–18. doi: 10.3390/su11164434

[40] Du Y, Zhou H, Yuan Y. B, Liu X. Explore knowledge-sharing strategy and evolutionary mechanism for integrated project team based on evolutionary game model. Advances in Civil Engineering. 2019; 1–23. doi: 10.1155/2019/4365358

[41] Do Leeuw T, Gossling T. Theorizing change revisited: an amended process model of institutional innovations and changes in institutional fields. Journal of Cleaner Production. 2016; 135: 435–448. doi: 10.1016/j.jclepro.2016.06.119

