# Supplementary material for: How to alter path dependency and promote the use of EPC model in public projects of China?
Source: PLoS One. 2022 Apr 19;17(4):e0266957. doi: 10.1371/journal.pone.0266957 (PMC9017937; doi:10.1371/journal.pone.0266957)
Supplement: S1 File — (DOCX) [file pone.0266957.s001.docx]

| Year | Number of EPC projects | Number of total projects | proportion |
| --- | --- | --- | --- |
| 2016 | 286 | 76440 | 0.003741497 |
| 2017 | 1290 | 241807 | 0.005334833 |
| 2018 | 4825 | 401471 | 0.012018303 |
| 2019 | 8098 | 473263 | 0.017110993 |
| 2020 | 10545 | 432729 | 0.0243686 |
| 2021 | 12109 | 517852 | 0.023383129 |
